# Secondary Sarcopenia and Spinal Cord Injury: Clinical Associations and Health Outcomes

**DOI:** 10.3390/jcm13030885

**Published:** 2024-02-02

**Authors:** Anamaria Gherle, Carmen Delia Nistor-Cseppento, Diana-Carina Iovanovici, Iulia Ruxandra Cevei, Mariana Lidia Cevei, Danche Vasileva, Stefania Deac, Dorina Stoicanescu

**Affiliations:** 1 Doctoral School of Biomedical Sciences, Faculty of Medicine and Pharmacy, University of Oradea, 410087 Oradea, Romania; bl_anamaria@yahoo.com (A.G.); stefaniacristea95@yahoo.ro (S.D.); 2 Department of Psycho-Neurosciences and Recovery, Faculty of Medicine and Pharmacy, University of Oradea, 410073 Oradea, Romania; cevei_mariana@yahoo.com; 3 Institute of Cardiovascular Diseases Timisoara, 13A Gheorghe Adam Street, 300310 Timisoara, Romania; iuliacevei@yahoo.com; 4 Faculty of Medical Sciences, Goce Delcev University, P5MX+HP6, 2000 Stip, North Macedonia; dance.vasileva@ugd.edu.mk; 5 Microscopic Morphology Department, “Victor Babes” University of Medicine and Pharmacy Timisoara, 300041 Timisoara, Romania; dstoicanescu@gmail.com

**Keywords:** spinal cord injury, sarcopenia, immobilization osteoporosis, spasticity

## Abstract

**Background:** Sarcopenia and spinal cord injury (SCI) often coexist, but little is known about the associations. This study aimed to assess the impact of SCI on muscle and bone mass and the correlations between the clinical characteristics of SCI patients and sarcopenia. **Methods:** A total of 136 patients with SCI admitted to rehabilitation hospital were included in this study. The type and severity of injury (AIS), level of spasticity (MAS), bone mineral density and Appendicular Lean Muscle Mass (ALM) were assessed. Sarcopenia was diagnosed according to EWGSOP2 cut-off points for ALM. **Results:** Subjects were divided into two groups: Group S-SCI (N = 66, sarcopenia group) and Group NS-SCI (N = 70, without sarcopenia). Mean ALM values in the two groups were 0.49 and 0.65, respectively. A total of 75% of women and 42.9% of men developed sarcopenia. The mean age was 35.8 years in the sarcopenic patients and 41.5 in the non-sarcopenia group. Over 55% of AIS Grades A and B cases, 69.7% of MAS level 0 cases and 51.6% of the patients with osteoporosis had sarcopenia. The mean number of comorbidities was 2.7 in the sarcopenia group. **Conclusions:** Gender, type of injury, presence of multiple comorbidities and age were directly associated with sarcopenia; meanwhile, surprisingly, spasticity level and the presence of immobilization osteoporosis were not.

## 1. Introduction

The progressive loss of muscle mass and strength, with the impaired physical performance of individuals and associated with advancing age, has been defined as sarcopenia [1,2]. The term was first introduced by Rosenberg (1989) [3].

The etiology is multifactorial. Age-related decline, chronic diseases, presbyphagia, qualitative and quantitative muscle tissue impairment, hormonal changes and cellular metabolism (the imbalance between protein synthesis and degradation) are some of the factors involved in the occurrence of sarcopenia [4,5]. The loss of muscle mass and strength associated with the ageing process defines primary sarcopenia, which may contribute to a decrease in mobility, balance, coordination and the ability to perform activities of daily living [6]. Secondary sarcopenia is defined by the association of ageing with other evident factors or comorbidities [7].

In 2010, the European Working Group on Sarcopenia in Older People (EWGSOP) met to develop diagnostic criteria. They developed clear diagnostic criteria and a globally accepted definition for age-related sarcopenia. The working definition until 2018 was that sarcopenia is a syndrome characterized by the concurrent presence of both low muscle mass and low muscle function or low physical performance [6]; however, in early 2018, the EWGSOP2 re-convened to update the original definition and establish new cut-off points for sarcopenia, with the aim of highlighting the latest emerging scientific and clinical evidence that has accumulated over the past decade [8]. The conclusion reached by Cruz-Jentoft et al. in 2019 was that it is common among older adults but can also occur earlier in life [8].

Osteosarcopenia is defined by the concomitant presence of osteopenia/osteoporosis and sarcopenia. The etiology of osteosarcopenia is multifactorial, involving several factors, both genetic and environmental. In addition, a poor nutritional status and lack of physical activity, such as prolonged immobilization, are key risk factors for osteosarcopenia [9].

Spinal cord injuries (SCI) are typically characterized by a loss of function (motor and/or sensory) distal to the lesion and include multiple impairments: paralysis of voluntary muscles, altered sensory function, mobility disorders and joint contractures, abnormal muscle tone, pain and cardio-pulmonary deconditioning [10]. Road accidents are the most frequent causes of SCI, followed by falls resulting in bone fractures, gunshot wounds and sports. Several secondary long-term complications can occur after SCI, such as respiratory and cardiovascular complications, urinary and bowel complications, spasticity, bone disease and last but not least, loss of the skeletal muscles [11,12,13].

The loss of motor and/or sensory function leads to a reduction in physical activity which favors deconditioning [7]. Muscle unloading, lack of voluntary contraction, spasticity and injuries to small vessels cause changes in muscle fibers and histochemical changes in the muscle cells.

Patients with complete SCI can lose 20–55% of their muscle mass, while those with incomplete damage lose between 20 and 30% of their muscle mass [14].

The working definition of sarcopenia in patients with SCI is, to our knowledge, still not clear, and the current application of the definitions of sarcopenia in subjects with SCI requires further research [7].

The aim of this study was to evaluate the impact of SCI on muscle and bone mass, depending on a series of clinical characteristics of SCI patients.

## 2. Materials and Methods

### 2.1. Study Design

A cohort study was conducted from 2019 to 2022. Patients from the “Băile Felix Medical Rehabilitation Clinical Hospital” diagnosed with SCI were enrolled in this study. The study was approved by the local Ethics Committee (4016/30.04.2018) and was conducted in accordance with the principles of the Declaration of Helsinki. All participants provided their written consent before participating in this study.

### 2.2. Inclusion/Exclusion Criteria

Inclusion criteria were: patients with an established diagnosis of SCI from traumatic causes, aged 18 to 75 years, and at least 6 months since the traumatic event. Exclusion criteria for this study were: age under 18 years, SCI due to non-traumatic causes such as degenerative cervical myelopathy, tumors, birth defects, disruption of the blood supply to the spinal cord, multiple sclerosis, amyotrophic lateral sclerosis, infections and patients who could not undergo whole body dual X-ray absorptiometry (DXA) scanning.

### 2.3. Study Tools

**Figure 1:**
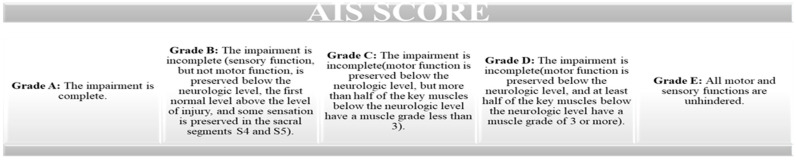
American Spinal Injury Association Impairment scale (AIS).

This study used the revised ASIA scale (proposed by the American Spinal Injury Association) to assess the severity of SCI. This scale evaluates the motor score (by assessing key muscles) and the sensory score, by testing tactile and pain sensitivity such as a light touch and a pin prick (on dermatomes) (Figure 1) [15].

**Figure 2:**
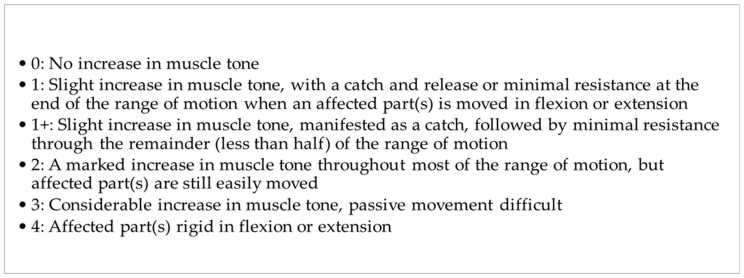
The modified Ashworth Scale (MAS).

The level of spasticity was assessed using the MAS [16]. The original Ashworth Scale is a numerical scale from 0 to 4. Lower scores indicate normal muscle tone, and higher scores represent spasticity [17]. The MAS adds 1+ to the scale to increase sensitivity (Figure 2) [18].

Bone mineral density was determined using DXA scans for all patients (Medix 90, Medilink Sarl, France). Skeletal muscle mass index (SMI) (appendicular skeletal muscle (ASM) to height^2^: SMI = ASM/h^2^; kg/m^2^) [19] or ALM was determined via full-body DXA using whole body assessment. We established the diagnosis of sarcopenia according to the cut-off value recommended by EWGSOP2 for ALM. This cut-off value was 0.54. DXA indicates the total amount of lean tissue but does not measure muscle mass. ALM, derived from DXA scans, is the sum of lean tissue in the arms and legs. ALM alone or scaled to squared height (ALM/height^2^) or body mass index (ALM/body mass index), was the most common parameter used as a proxy for muscle mass in our sarcopenia study [19]. Immobilization osteoporosis diagnosis was established using the Z-score for the lumbar spine and right and left hip via DXA, and according to the EWGSOP, the Z-score cut-off value is −1.5 [20].

**Figure 3:**
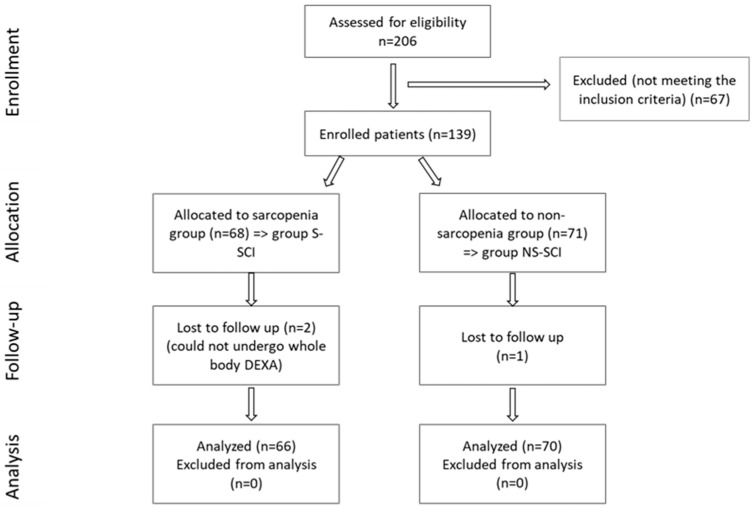
CONSORT flow diagram of the present study.

A total of 206 SCI patients were recruited, as can be seen in the CONSORT flow chart shown in Figure 3, but only 136 met the inclusion criteria. After determining the ALM value, subjects were divided into two groups, according to the ALM values:

- Group S-SCI, which included 66 patients with SCI and ALM above the cut-off values (sarcopenia group).
- Group NS-SCI, which included 70 patients with SCI and ALM below the cut-off values (without sarcopenia).

The period of time since the SCI and the evaluation moment was calculated in months.

### 2.4. Sample Size

To calculate the sample size, we used the total number of patients admitted with a diagnosis of SCI (according to the inclusion criteria) during the period established in this study. The calculation formula that was used in this study to calculate the minimum sample was n = t^2^ pq/(x^2^ + t^2^ pq/N), where *p* is the probability of occurrence of the phenomenon, q is the counter-probability, q = 1 − *p*, t is the probability factor, x is the error limit, N is the community volume. The value of n is maximum if the product of pq is maximum (*p* = q = 0.5). The 95% probability corresponds to a value of t = 1.96. A limiting error of 0.1 was set. If N is large, above 10,000 (in our case N = 269), the ratio t^2^ pq/N is neglected. Applying the above formula yielded N = 96; the formula applies to studies where the target characteristic is an alternative.

### 2.5. Statistical Analysis

All statistical analysis was generated using the JASP software, version 0.18.0. The calculation of the *p*-values was performed using Student’s *t*-test and the Mann–Whitney U test for numerical variables. The Poisson regression was used for count data and the chi-square test for nominal and ordinal variables. For accurate results, we conducted multiple comparisons; to control the false-positive rate, we conducted the Bonferroni correction when assessing the statistical significance of the results: we compared the obtained *p*-values with α* = 0.05/k, where k is the number of tests conducted.

### 2.6. Study Hypotheses

Given the latest research on sarcopenia and the fact that sarcopenia and SCI often coexist, but little is known about the associations between these two pathologies, our study aimed to assess the impact of SCI on muscle and bone mass and the correlations between a series of clinical characteristics of SCI patients, such as the neurological level of injury (NLI), the type of lesion (complete versus incomplete), AIS grade, level of spasticity measured with the MAS, period of time since the traumatic event and presence of complications and the prevalence of sarcopenia in these patients.

We hypothesized that more men than women would have SCI, according to the epidemiological studies in the literature [10], and that more women than men would be susceptible to having sarcopenia [21]. We also presumed that patients with a complete lesion would be more susceptible of developing sarcopenia versus patients with an incomplete lesion. We assumed that the presence of immobilization osteoporosis is directly related to the risk of developing secondary sarcopenia in patients with SCI, based on our clinical observations and experience, but also based on the literature review [20,21,22]. Regarding the period of time elapsed since SCI, we hypothesized that the mean time since injury was longer for the sarcopenia patients compared to those without sarcopenia [23].

## 3. Results

### Sample Characteristics

The anthropometric data, number of months since SCI, NLI, AIS grade, MAS score, comorbidities, Z-score for immobilization osteoporosis, mean ALM values for both study groups (Group S-SCI, Group NS-SCI) are presented in Table 1. Mean ALM values in the two study groups were: 0.49 in the S-SCI group, and 0.65 in the NS-SCI group. A total of 48.52% of SCI patients were diagnosed with sarcopenia, while 51.47% were not.

A total of 75% of women were diagnosed with sarcopenia. On the other hand, 42.9% of men were diagnosed with sarcopenia. A chi-square test in each gender group was performed to test these differences. Multiple comparisons were performed; therefore, the Bonferroni correction was used to control the false-positive rates: we compared the obtained *p*-values with α* = 0.05/k, where k is the number of tests conducted (k = 2 in this case). As the chi-square tests show, women are more likely to be diagnosed with sarcopenia (*p* < 0.025), whereas among men, these proportions do not seem to differ significantly. The computed log odds ratio showed that women were approximately 1.4 times (40%) more likely to be diagnosed with sarcopenia than men.

The mean age of the sarcopenic patients was lower than the mean age of the patients without sarcopenia (35.8 years and 41.5 years, respectively).

Descriptive statistics in Table 1 revealed that among the patients with a cervical (C)-level injury slightly more were diagnosed with sarcopenia (52.9%). Among the cases with a lumbar (L)-level injury, 40% of the patients were diagnosed with sarcopenia. Finally, among the patients with a thoracic (T)-level injury, slightly fewer were diagnosed with sarcopenia (47.1%). To test whether the frequency distributions of the SCI groups were similar across the lesion levels groups, we conducted a chi-square test within each lesion level group. Bonferroni correction was also used as we conducted multiple comparisons, to control the false-positive rate: we compared the obtained *p*-values with α* = 0.05/k, where k is the number of tests conducted (k = 3 in this case). The results are presented in Table 1. The data showed that the prevalence of sarcopenia did not vary across the three lesion level groups, as *p* > 0.017.

Descriptive statistics in Table 1 also showed that among the patients with AIS Grade A, slightly more were diagnosed with sarcopenia (55.8%). Of the patients with AIS Grade B, 59% were diagnosed with sarcopenia. Among the patients with AIS Grade C, 28.6% were diagnosed with sarcopenia, while 35.3% of the patients with AIS Grade D were diagnosed with sarcopenia. We conducted a chi-square test within each AIS grade group to test whether the frequency distributions of the SCI groups are similar across the AIS grade groups. As we conducted multiple comparisons, and to control the false-positive rate, we conducted the Bonferroni correction: we compared the obtained *p*-values with α* = 0.05/k, where k is the number of tests conducted (k = 4 in this case). The results are presented in Table 1. We did not find any statistically significant differences in the proportions of S-SCI and NS-SCI patients across the AIS grade groups (*p* > 0.0125).

Regarding the level of spasticity, our data showed that 69.7% of the patients with MAS level 0 were diagnosed with sarcopenia. Among patients with MAS level 1, 42.9% were diagnosed with sarcopenia, while 57.1% were not. Among patients with MAS level 2, 41.5% were diagnosed with sarcopenia and among those with MAS level 3, 40.7% had sarcopenia. Finally, among the patients with a level 4 on the MAS, 42.9% were diagnosed with sarcopenia. To test whether the frequency distributions of the SCI groups were similar across the Ashworth level groups, we conducted a chi-square test within each Ashworth level group.

We conducted the Bonferroni correction: we compared the obtained *p*-values with α* = 0.05/k, where k is the number of tests conducted (k = 5 in this case), as we conducted multiple comparisons and also to control the false-positive rate. We did not find any statistically significant differences in the proportions of S-SCI and NS-SCI patients across the Ashworth level groups (*p* > 0.01).

**Figure 4:**
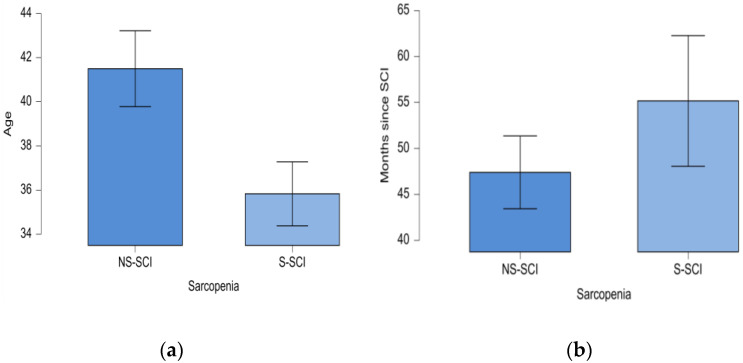
(**a**) Mean age (years) of patients in the two groups: NS-SCI and S-SCI. (**b**) Mean time (months) since SCI in the two groups: NS-SCI and S-SCI.

The minimum age in the NS-SCI group was 20 years, while in the S-SCI group it was 18 years; the maximum age in the NS-SCI group was 73 years, while in the S-SCI group it was 67 years. Statistical significance of these differences was tested conducting the *t*-test for independent samples. The results showed that patients with sarcopenia were significantly younger than those without sarcopenia, *p* < 0.05, and the effect size was medium (d = 0.431) (Figure 4a).

The mean time since the SCI was 55 months (ranging from 2 months to 312 months) in the sarcopenia group and about 47 months (ranging from 12 months to 132 months) in the non-sarcopenia group. The statistical significance of this difference was tested using the non-parametric Mann–Whitney U test, because the normality and equality of variances assumptions were not met by our data. According to the Mann–Whitney U test, our data do not support the hypothesis that the mean time since the SCI is longer for the sarcopenia patients compared to those without sarcopenia (W = 2379, *p* > 0.05, Figure 4b).

Data presented in Table 2 reveal that the mean Z-scores for the lumbar area (lumbar Z-score), the right hip (right hip Z-score) and the left hip (left hip Z-score) tend to be lower in the S-SCI group compared to the NS-SCI group.

Moreover, 41% of the patients without osteoporosis were diagnosed with sarcopenia. Among the patients with osteoporosis, 51.6% were diagnosed with sarcopenia. We conducted a chi-square test within each group, to test whether the frequency distributions of the SCI groups were similar across patients with or without osteoporosis. The Bonferroni correction was used as we performed multiple comparisons, and also to control the false-positive rate: we compared the obtained *p*-values with α* = 0.05/k, where k is the number of tests conducted (k = 2 in this case). The results are presented in Table 3.

We did not find significant differences in the frequency distributions of immobilization osteoporosis across the groups of patients with and without sarcopenia (*p* > 0.025).

**Figure 5:**
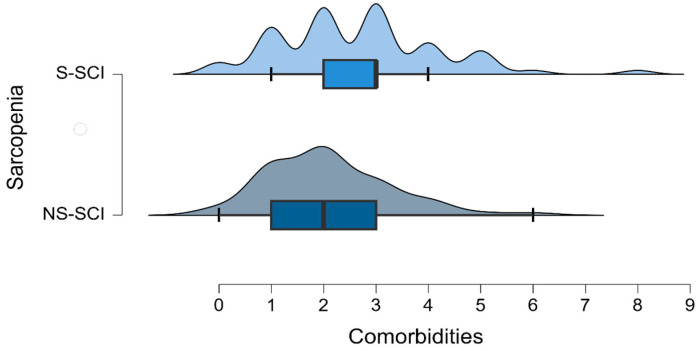
Density plot of the number of comorbidities across the groups of patients with and without sarcopenia.

The mean number of comorbidities was 2 in the non-sarcopenia group (ranging between 0 and 6) and 2.7 in the sarcopenia group (ranging between 0 and 8) (Figure 5). The most common comorbidity in each group was hypertension. Among the patients without hypertension, the number of sarcopenic and non-sarcopenic patients was almost the same (50.4% versus 49.6%). A total of 30.8% of the patients with hypertension were diagnosed with sarcopenia. To test whether the frequency distributions of the SCI groups were similar across patients with or without hypertension, we conducted a chi-square test within each group. We conducted the Bonferroni correction: we compared the obtained *p*-values with α* = 0.05/k, (k = number of conducted tests, two in this case). We did not find a statistically significant association between sarcopenia and the presence of hypertension (*p* > 0.025).

Poisson regression (also known as a log-linear model) was used to test the association between sarcopenia and the number of comorbidities. The deviance goodness-of-fit test indicates that the model fits the data (D (134) = 107.858, *p* = 0.953). The tables below (Table 4 and Table 5) show the model summary and the model coefficients.

The model summary table shows that there was a significant effect of sarcopenia on the number of comorbidities that patients were diagnosed with [χ^2^ (1) = 4.843, *p* < 0.05]. Thus, the coefficients table reveals that the number of comorbidities is 1.279 times higher in patients with sarcopenia than in those without the disease. In other words, there is a 27.9% increase in the number of comorbidities among patients with sarcopenia compared to those without this condition.

## 4. Discussion

The goal of this study was to assess the impact of SCI on muscle and bone mass, and the correlations between different clinical characteristics of SCI patients and the presence of sarcopenia in these cases.

SCI is a severe and disabling disease, leading to loss of innervation of skeletal muscles, decreased motor function and significantly reducing the load on skeletal muscles, all these leading to atrophy. Skeletal muscle atrophy is accelerated by fractures, hormone level fluctuation, inflammation and oxidative stress damage. Muscle atrophy leads to impaired skeletal muscle function around and below the injury site [24]. In SCI, the inability or decreased ability to perform physical exercise are often associated with sarcopenia [25,26,27]. Leone et al. stated that neurogenic factors, musculoskeletal disuse and cellular/molecular events contribute to more rapid and debilitating levels of muscle and bone loss in individuals with SCI. The absolute causes of bone loss are not yet known; however, sarcopenia could be one of the causes [28]. Dionyssiotis et al. also considered that the pathophysiology of sarcopenia in SCI is complex. Although muscle mass does not predict muscle strength or physical performance, it is significantly correlated with these parameters and contributes to disability and frailty in old people. There are no guidelines or even recommendations regarding sarcopenia in SCI. Proper measurements of performance in SCI are not yet available [7]. Persistent neuromuscular paralysis leads to muscle atrophy related to both neurologic injury and functional immobility. Neuromuscular paralysis is one of the secondary causes of muscle atrophy and sarcopenia. Lean muscle mass has been correlated with strength and functional performance in healthy children, being considered a strong predictor of health and performance in all individuals. Muscle mass is frequently used as a surrogate for muscle strength, especially in young children [29,30].

The cross-sectional area of the skeletal muscle (which represents approximately 40% of the body weight) decreases fast in the following 1–17 months after a trauma. Muscular atrophy in patients with SCI is partially explained by the rapid changes in the quality of muscle proteins, activating proteolytic enzymes and proteases from mitochondria, the increase in the production of reactive oxygen species and the decrease in oxidative capacity [31,32]. Based on a previous study, Ishimoto et al. used only ASM as the criterion for identifying patients with sarcopenia. Their study revealed two clinical observations. Sarcopenia, obesity and sarcopenic obesity were prevalent among individuals with SCI and female gender, tetraplegia, motor-complete injury and inability to walk were identified as risk factors for muscle atrophy comparable to sarcopenia in persons with SCI [33]. Gater DR Jr et al. also stated that physiological changes occurring in the adipose tissue in SCI cases should be characterized as neurogenic obesity due to an obligatory sarcopenia, neurogenic osteoporosis, neurogenic anabolic deficiency, sympathetic dysfunction and blunted satiety associated with SCI [34]. According to these data, we expected to diagnose a high percentage of patients with sarcopenia, but, to our surprise, of all SCI cases, over 51% did not have sarcopenia. However, for the other 48.52% that were diagnosed with sarcopenia, the possible clinical implications that this condition raises are worth considering. The risk of frequent falling and the reduced ability or inability of a person with SCI to redress from a fall and lift from the floor, as well as other complications (e.g., cardiovascular disease occurrence, pulmonary disease) are crucial in patients with SCI. Sarcopenia synergistically worsens the adverse effects of the motor deficit in patients with SCI, leading to unfavorable health conditions, such as an increased risk of frailty and falls [35,36]. It can interfere with the capacity of a SCI patient to remain to some degree functional and can even influence the return of a SCI patient to a healthy, fulfilling independent lifestyle, which is the goal of every rehabilitation program. Therefore, the identification of modifiable risk factors associated with sarcopenia is of the utmost importance in order to prevent these adverse changes.

Comparing our results with the prevalence of sarcopenia in the general population, according to a meta-analysis of general population studies, published in 2017 by G Shafiee et al. the rate was 10% (95% CI: 8–12%) in men and 10% (95% CI: 8–13%) in women, respectively [37]; therefore, the prevalence of sarcopenia was high.

Comparing the two groups of patients with SCI (Group S-SCI and Group NS-SCI), there were statistically significant differences between the two study groups regarding gender, age and presence of comorbidities. Our results revealed that 75% of women suffering from SCI were diagnosed with sarcopenia. This finding was in line with the results of a study published by Hwang and Park (2022, N = 2697) that examined the prevalence of sarcopenia according to gender in young-old adults, reporting a higher prevalence of sarcopenia in females [38]. Ishimoto et al. [33], in a retrospective analysis (2023, N = 97), reported that female gender was a risk factor for sarcopenia. On the contrary, Zhang et al. in their published meta-analysis (2021) revealed no statistically significant difference in the pooled prevalence of sarcopenia in patients with heart failure between men and women [39]. Analyzing the effect of postoperative muscle loss and surgery-induced sarcopenia on the long-term outcomes of patients with gastric cancer, other authors found significant differences between genders, with more men having a rapid decrease in muscle mass, which impacted the overall survival [40].

However, our study revealed that women with SCI are more susceptible to developing sarcopenia. This could be due to the considerable sex-based differences in human skeletal muscle gene expression that regulates muscle mass, fiber composition and contractile function [41]. Considering the mentioned genetic traits, it is more likely that women would develop sarcopenia compared to men, androgens having a powerful anabolic effect that promotes muscle regeneration, while estrogen has muscle-protective effect through anti-inflammatory pathways that inhibit proteolysis [42]. On the other hand, the number of male patients was significantly higher compared to the number of female patients participating in this study, in agreement with previous studies [33]. Our results showed a significant association between gender and the likelihood of having sarcopenia. Despite the small sample size of female patients with SCI, they were more likely to develop sarcopenia. The computed log odds ratio showed that women with SCI are approximately 1.4 times (40%) more likely to be diagnosed with sarcopenia, which reinforces and strengthens the hypothesis issued at the beginning and is also in agreement with earlier observations [18]. A total of 42.9% of men were diagnosed with sarcopenia. Studies investigating muscle mass or sarcopenia and its correlation with the functional status of patients with SCI are few and at infrequent intervals. Previously, the somewhat few studies have shown that sarcopenia, obesity and sarcopenic obesity were prevalent among individuals with SCI; female gender, level of injury (tetraplegia) and a complete injury leading to inability to walk (AIS A, B) were identified as risk factors for sarcopenia in individuals with SCI [19,33].

The prevalence of sarcopenia may vary depending on the diagnostic criteria, definitions, study group, anthropometric values and measured parameters [8]. We need to be aware that we cannot perform a walking test with patients with disabilities, tetraplegia or paraplegia. We also lack the proper measurement of strength, assessed with hand dynamometers, because patients with tetraplegia cannot perform the handgrip test and for those with paraplegia it would be very difficult or even impossible, depending on the NLI. Therefore, it would be biased, not to mention the fact that patients with paraplegia who use a wheelchair may develop stronger upper limbs through the indirect training effect of their daily wheelchair activities.

Our results also revealed that patients with sarcopenia were significantly younger than those without sarcopenia. Primary sarcopenia is an age-related process associated with a loss of muscle mass [43,44]. This does not fit into the general frame of our study design, because patients with SCI participating in this study were, in general, young adults, with a mean age of 41 years in the NS-SCI group and 35 years in the S-SCI group; this reinforces the above mentioned hypothesis, and aligns with epidemiological studies regarding the average age at which SCIs occur, which was 37.6 years in a study from 2000 [45]. Another study published in 2023 by Tanaka et al. (N = 1039) found a statistically significantly higher mean age in the sarcopenic group compared with the non-sarcopenic one [46]. However, secondary sarcopenia occurs if other factors, besides aging, are evident in the study sample, as per our research [47].

Paradoxically, we did not find any significant correlations between the presence of immobilization osteoporosis and sarcopenia, which we would have expected, as the prevalence of secondary osteoporosis in SCI is high [48]. The presence of sarcopenia was found to be associated with reduced bone mineral density and osteoporosis [49]. Yoshimura et al., in a study published in 2017, examined the relationship between osteoporosis and sarcopenia (diagnosed according to AWGS criteria) based on the results of a survey of bone mineral density in 1099 participants and determined an osteoporosis prevalence of 24.9%. In addition, although 18.9% of these patients had sarcopenia, the prevalence of sarcopenia in the study group was 8.2% [50]. The prevalence of secondary conditions among patients with SCI depends on a variety of aspects; the severity and completeness of the injury are some of the most important ones. Gianna Rodriguez et al., in a study published in 2021 (N = 9081), investigated the risk of developing musculoskeletal comorbidities by comparing 9081 privately insured patients with 1,474,232 adults without SCI, and the results showed that adults with SCIs have a significantly higher incidence and risk for musculoskeletal comorbidities, as compared to adults without SCIs [43]. However, in our study, despite the lack of statistical significance, an important percentage of patients (46.84%) from the S-SCI group were diagnosed with secondary osteoporosis.

Considering the MAS scores, we expected that patients with a high degree of spasticity (who scored 3 or 4) would be less susceptible of developing sarcopenia. According to a study published in 2022 by Li et al. (N = 28) about sarcopenia following stroke, the authors demonstrated that spasticity in stroke survivors actually had a protective role against muscle loss in the lower limbs [51,52].

Many of the long-term outcomes of SCI are related to muscle and bone loss due to immobilization. The impact of SCI can vary depending on the NLI, but also on the type of lesion (complete/incomplete). There are short-term impacts and long-term impacts, as Leone et al. highlighted in a review article published in September 2023. Long-term impacts include sarcopenia and osteoporosis [28].

The last statistically significant difference between our study groups was the presence of comorbidities. On average, patients without sarcopenia had approximately 2 comorbidities associated with SCI, while patients with sarcopenia had approximately 2.7 comorbidities associated with SCI. Sarcopenia can be considered itself a comorbidity and is often studied in numerous clinical trials, and it seems that the presence of sarcopenia is associated with other secondary conditions such as respiratory disease, diabetes, dementia and cardiovascular disease [53].

Secondary health conditions affecting the sensory, respiratory, cardio-vascular, genitourinary and tegumentary system impact a SCI patient’s life, being debilitating and potentially life-threatening [54]. Tallqvist et al. assessed the comorbidities and secondary health conditions (SHCs) among the Finnish population with SCI (2022, N = 884) and found that the prevalence of comorbidities and SHCs was common among elderly persons with SCI, with age being the strongest predictor for multimorbidity [55].

In our research, the most common comorbidity in both groups was hypertension; however, we did not find a statistically significant association between sarcopenia and the presence of hypertension. SCI instead can cause a variety of physiological changes. The sudden reduction in physical activity in individuals with SCI is translated into elevated risk of cardiovascular diseases; therefore, they are among the leading causes of death in the SCI population [56].

Kepler et al., in a retrospective case–control study published in 2015 (N = 92), investigated the effects of pre-existing hypertension in a number of patients with acute SCI and they found that chronic hypertension was an independent risk factor for poor outcomes in patients with acute SCI. This finding was independent of age and other comorbidities [57].

### 4.1. Strengths and Limitations of the Study

This is the first study in Romania, to our knowledge, that has evaluated sarcopenia in SCI patients and its correlation with gender, age, time elapsed since the onset of the disease, injury level and muscle tone. An important aspect to consider is the large number of patients recruited and evaluated.

Our study had certain limitations: (1) sarcopenia was diagnosed using only the ALM score, because some patients were tetraplegic and we could not use the Jamar dynamometer to determine muscle strength, and we also could not perform a walking test for these patients; the sarcopenia questionnaire, such as SARC-F [54], also could not be used; (2) the question remains as to whether the cut-off points for unaffected and able-bodied populations can be applied to SCI patients; (3) SCI with its pathophysiology may influence the validity of the results.

### 4.2. Future Directions

Future research is needed to establish clear definitions of sarcopenia in SCI patients with disabilities. Following SCI, many patients develop motor, but probably most importantly, functional impairments that can affect their daily living and reduce the quality of life [54]. In addition, in the chronic phase, multiple complications can occur. One of them can be secondary sarcopenia; the rehabilitation goals during this phase are to prevent further damage from the injury and to slow down or reverse the process of muscle loss. The general recommendation for the management of sarcopenia is to follow a resistance-based exercise program and to follow a high-protein diet [47].

There are several pharmacologic approaches to prevent or reduce muscle atrophy after SCI. Testosterone can increase muscle mass and strength in older individuals and a meta-analysis has confirmed its safety [55]. Acteoside-treated muscles had a significantly greater mass when compared to the vehicle treatment, which suggests that acteoside promotes skeletal muscle recovery and regeneration, potentially independently of exercise-induced myokine secretion [56]. Estrogen can also play a role in remodeling the extracellular matrix and muscle fiber expansion after unloading or not being used [57]. Imagery-based rehabilitation might be a promising therapeutic approach in patients with SCI [58].

The number of patients suffering from SCI increases year after year, and so do the secondary complications that can occur alongside it. The search for therapeutic modalities to improve the quality of patients’ lives with SCI continues.

## 5. Conclusions

The study results showed that patients with sarcopenia were significantly younger than those without sarcopenia. The following clinical features of SCI patients: gender, the type of injury, the presence of multiple comorbidities and age were directly associated with sarcopenia, while, surprisingly enough, spasticity level and the presence of immobilization osteoporosis were not proved to be associated with sarcopenia. There was a statistically significant association between the ALM value and the gender, considerably more women with SCI developing sarcopenia. The number of male patients was significantly higher compared to the number of female cases participating in this study, but fewer men with SCI had sarcopenia. Optimizing muscle mass should be an important objective in the management of SCI patients.

## Figures and Tables

**Table 1:** Baseline characteristics of the groups.

Parameter	Group S-SCI	Group NS-SCI	*p*-Value	SS/NS
ALM-value, M, SD	0.49 ± 0.042	0.65 ± 0.099	-	-
Patients N (%)	66 (48.529)	70 (51.471)	-	-
Age, M, SD (years)	35.83 ± 11.75	41.50 ± 14.36	0.013 *	SS
Female, N (%)	18 (75.00)	6 (25.00)	0.014 **	SS
Male, N (%)	48 (42.86)	64 (57.14)	0.131 **	NS
Number of months since SCI (M, SD)	55.17 ± 57.77	47.40 ± 33.17	0.620 ***	NS
Neurological level of injury				
Level Cervical, N (%)	27 (52.94)	24 (47.06)	0.674 **	NS
Level Thoracal, N (%)	33 (47.14)	37 (52.85)	0.633 **	NS
Level Lumbar, N (%)	6 (40)	9 (60)	0.439 **	NS
AIS Scale				
Grade A, N (%)	29 (55.77)	23 (44.23)	0.405 **	NS
Grade B, N (%)	23 (58.97)	16 (41.02)	0.262 **	NS
Grade C, N (%)	8 (28.57)	20 (71.43)	0.023 **	NS
Grade D, N (%)	6 (35.29)	11 (64.70)	0.225 **	NS
Modified Ashworth Scale				
Score 0, N (%)	23 (69.69)	10 (30.30)	0.024 **	NS
Score 1, N (%)	9 (42.85)	12 (57.14)	0.513 **	NS
Score 2, N (%)	17 (41.46)	24 (58.53)	0.274 **	NS
Score 3, N (%)	11 (40.74)	16 (59.25)	0.336 **	NS
Score 4, N (%)	6 (42.85)	8(57.14)	0.583 **	NS
Comorbidities (M, SD)	2.667 ± 1.522	2.086 ± 1.164	0.028 **	SS
Immobilization Osteoporosis, Z-score				
Lumbar M, SD	−1.318 ± 1.182	−1.280 ± 0.976	-	
Right hip M, SD	−1.948 ± 0.945	−1.444 ± 1.454	-	
Left hip M, SD	−1.802 ± 1.330	−1.467 ± 1.233	-	

M: mean value; SD: standard deviation value; N: number of the patients; Group NS-SCI: patients with SCI, without sarcopenia; Group S-SCI: patients with SCI, and sarcopenia; AIS scale—American Spinal Injury Association Impairment Scale *p* values statistical significance (*, Student’s *t*-test; **, chi-square test; *** Mann–Whitney U test); NS—without statistical significance; SS—statistically significant.

**Table 2:** Descriptive statistics for Z-scores across the S-SCI and NS-SCI groups.

Region Z-Score	Group	M ± SD	MIN	MAX
Lumbar Z-score	Group NS-SCI	−1.280 ± 0.976	−3.500	1.400
	Group S-SCI	−1.318 ± 1.182	−4.600	1.900
Right hip Z-score	Group NS-SCI	−1.444 ± 1.454	−3.700	4.400
	Group S-SCI	−1.948 ± 0.945	−3.900	−0.100
Left hip Z-score	Group NS-SCI	−1.467 ± 1.233	−3.700	2.800
	Group S-SCI	−1.802 ± 1.330	−3.900	5.200

M: mean value; SD: standard deviation value; Group NS-SCI: patients with SCI, without sarcopenia; Group S-SCI: patients with SCI, and sarcopenia; MIN: minimum value of Z-score; MAX: maximum value of Z-score.

**Table 3:** Frequency of immobilization osteoporosis in the S-SCI and NS-SCI groups.

Immobilization Osteoporosis	Group NS-SCI	Group S-SCI	*p*-Value **
Yes, N (%)	47 (48.45)	50 (51.55)	0.761 ** NS
No, N (%)	23 (58.97)	16(41.03)	0.262 ** NS

N: number of the patients; Group NS-SCI: patients with SCI, without sarcopenia; Group S-SCI: patients with SCI, and sarcopenia ; *p*-value statistical significance (**, chi-square); NS—without statistical significance.

**Table 4:** Model summary for the number of comorbidities.

Model	Deviance	AIC	BIC	df	Χ^2^	*p*-Value **
H₀	112.701	465.175	468.088	135		
H₁	107.858	462.332	468.157	134	4.843	0.028

H₀: the null hypothesis; H₁: the alternative hypothesis; Χ^2^: chi-square test; *p*-values statistical significance (**, chi-square); AIC: Akaike information criterion (AIC); BIC (Bayesian information criterion).

**Table 5:** Model coefficient for the number of comorbidities.

Coefficients						95% Confidence Interval for B	
	B	Std. Error	Exp(B)	Z-Score	*p*-Value **	Lower Bound	Upper Bound
Intercept	0.735	0.083	2.086	8.882	<0.001	0.568	0.893
Sarcopenia (Yes)	0.246	0.112	1.279	2.195	0.028	0.027	0.466

*p*-values statistical significance (**, chi-square); Std. Error: standard error; B: regression coefficient.

## Data Availability

Data of the patients are available in the medical archive of the hospital.
